# Prevalence of antibodies against visceralizing *Leishmania* spp. in brown rats from Grenada, West Indies

**DOI:** 10.14202/vetworld.2018.1321-1325

**Published:** 2018-09-24

**Authors:** Alexa Rosypal von Dohlen, Nautica Cheathem, Keshaw Tiwari, Ravindra Nath Sharma

**Affiliations:** 1 Department of Natural Sciences and Mathematics, College of STEM, Johnson C. Smith University, Charlotte, NC, USA; 2 Department of Pathobiology, School of Veterinary Medicine, St. George’s University, Grenada, West Indies

**Keywords:** brown rats, Grenada, leishmaniasis, prevalence, serum antibodies

## Abstract

**Background and Aim:**

*Leishmania* spp. are known to cause disease in man and animals. Rats are considered important reservoir hosts and transmission takes place through the bite of female sand fly, *Phlebotomus* spp. To the best of our knowledge, there is no published information on *Leishmania* infection in rats in Grenada. This study was conducted to estimate the antibodies for visceralizing *Leishmania* spp. (VL) in rats (*Rattus norvegicus*) from Grenada.

**Materials and Methods:**

A total of 146 brown rats (*R. norvegicus*) were trapped live from two parishes (St. George and St. David) in Grenada. Following anesthesia, blood was collected from the heart through thoracic puncture. The serum was collected after the centrifugation of blood. Serum was tested for antibodies to VL. with a commercially available immunochromatographic dipstick test which is licensed for use in animals and humans.

**Results:**

The seroprevalence of antibodies against *Leishmania* spp. was found in 34 of 146 rats (23.3%; CI 95% from 16.70 to 30.99). No significant differences were found between sexes and young or adults. The prevalence between parishes (St. George and St. David) was also not significant.

**Conclusion:**

The results show that rats (*R. norvegicus*) in Grenada are exposed to *Leishmania* spp. The rats could play an important role in the transmission of leishmaniasis to humans and other animals in Grenada.

## Introduction

Leishmaniasis is a wide-ranging spectrum of diseases caused by parasites in the genus *Leishmania*. *Leishmania* spp. are insect-vectored intracellular protozoan parasites causing disease in humans and animals [1]. The clinical manifestations of leishmaniasis usually depend on the species of *Leishmania* infecting the host. Parasites in the *Leishmania* *donovani* complex typically cause visceral leishmaniasis (VL), which leads to hepatosplenomegaly and can be fatal if left untreated. Cutaneous leishmaniasis is commonly caused by infection with *Leishmania major* and *Leishmania tropica*, whereas the mucocutaneous form is caused by *Leishmania braziliensis* [2]. Dogs are considered the main reservoir of zoonotic species of *Leishmania*, including the *L. donovani* complex. In addition to dogs, however, many other wild animals are considered reservoir hosts such as wild canids, primates, and rodents [3].

Leishmaniasis is transmitted by the bite of female phlebotomine sand flies [4]. Flagellated promastigotes are injected in the skin of the host while the sand fly blood feeds. Once inside the host, promastigotes penetrate into macrophages and transform into spherical, non-flagellated amastigotes. Amastigotes multiply within macrophages of the host by binary fission. Eventually, infected macrophages burst open and release the amastigotes which may move to internal organs in the case of VL. Sand fly vectors ingest amastigotes while obtaining a blood meal from the infected host. Inside the insect vector, amastigotes change to promastigotes in the intestine. Some promastigotes attach to the epithelium of the foregut and others remain free and are transmitted to a susceptible host through the bite wound.

Leishmaniasis has been reported in the intertropical zones of Africa and America and into the temperate zones of South America, Europe, and Asia. Leishmaniasis is endemic in countries of South America, but little is known about leishmaniasis in neighboring Caribbean countries. Zeledon [5] reported sporadic cases of CL in humans from Martinique, Trinidad, and Guadeloupe. VL is rare in the Caribbean [5], but the first case of VL in Martinique was recently reported in an AIDS patient [6].

Little is known of *Leishmania* spp. infections in Grenada, West Indies, and no human cases of VL have been reported. Rosypal *et al*. [7] found no evidence of antibodies to VL parasites in 77 pet or stray dogs from Grenada. More recently, Kumthekar *et al*. [8] detected antibodies specific for VL in stray dogs (3.7%) and owned dogs (2.0%) which indicated that dogs in Grenada are exposed at a low level to VL.

In addition to dogs, rodents are also important reservoir hosts for leishmaniasis. In the Caribbean, rats are suspected to be animal reservoir [5,6,9,10], and rodent burrows are common breeding sites for phlebotomine sand flies [11].

To the best of our knowledge, there is no report of *Leishmania* spp. infection in rats from Grenada. The current research aimed to determine the exposure of rats (*Rattus norvegicus*) to VL. in Grenada.

## Materials and Methods

### Ethical approval

The project (detection of zoonotic pathogens in brown rats [*R. norvegicus*] in Grenada) was approved by the Institutional Animal Care and Use Committee (IACUC # 16009-R) of the St. George’s University, Grenada.

### Study area

**Figure-1:**
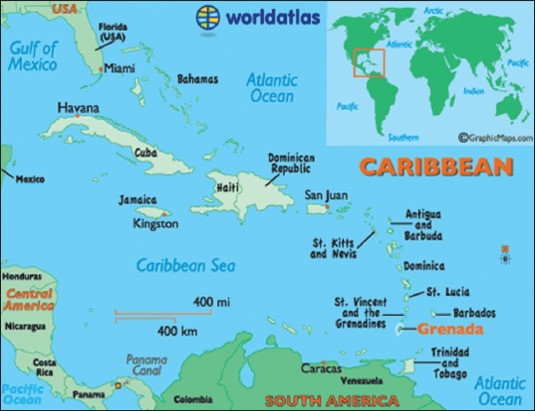
Map showing location of Grenada in the Caribbean.

**Figure-2:**
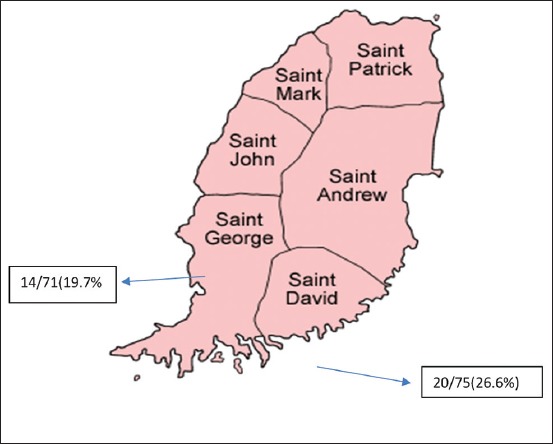
Grenada and parishes.

Grenada is the southernmost country in the Caribbean Sea with an area of 348.5 km^2^ (Figure-1). The country with low hills, small trees and shrubs, and tropical climate is most suitable for rats. The country is comprised six parishes: St. Patrick, St. Mark, St. Andrew, St. John, St. George, and St. David (Figure-2). St. David and St. George parishes, which have a higher human population compared to other four parishes, were selected for the study (Figure-2).

### Collection of rats

A total of 146 brown rats were collected live from May 1 to July 14, 2017, using traps (45 cm L ×15 cm W ×15 cm H) with cheese and various local fruits as bait. Attempts were made to trap the rats from and near the residential buildings. Traps were placed 2 days per week in the evening and visited next day morning. Traps with rats were covered with black cloth and transported to the necropsy laboratory of the School of Veterinary Medicine and transferred to the anesthesia machine. Rats were anesthetized using isoflurane in oxygen through portable vet anesthesia machine isoflurane vaporizer VET CE., manufacturer DRE (Avante Health Solution Company, USA.)

### Collection of samples

The anesthetized rats were examined for their physical health and weighed. Gender was also recorded. Rats <100 g were grouped as young and over 100 g as adult, following the methodology used by Panti-May *et al*. [12]. Blood was collected from the heart through the thoracic wall and rats were exsanguinated this way.

Sera were separated from the blood by centrifugation at 1500 g for 15 min at room temperature and stored at −80°C till tested.

### Serology

Rats were tested for antibodies to visceral *Leishmania* spp. with a commercially available immunochromatographic (ICT) dipstick test which is licensed for use in animals and humans (Kalazar Detect™ Canine Rapid Test, InBios International Ltd., Seattle, Washington). The qualitative ICT is based on recombinant antigen K39, an immunodominant amastigote protein, to detect antibodies specific to VL species [13]. The ICT has been used in previous studies of rodents for detection of antibodies to VL [14,15]. Rat sera were tested according to the manufacturer’s test procedure. Briefly, rat serum samples were thawed, vortexed, and 20 µL of serum was placed on the sample pad of the dipstick. The ICT was then placed vertically in the well of a 96-well plate, and three drops (~150 µL) of provided chase buffer were added to the base of the well to allow the serum to migrate up the dipstick. Results were collected in 10 min. The presence of a red or pink line in the control line and test line within the test area indicated a positive test. A single red or pink line in the control line area was a negative result. Antibody screening was performed at the Department of Natural Sciences and Mathematics, Johnson C. Smith University, Charlotte, North Carolina, USA.

### Statistical analysis

The data were analyzed by the Fisher’s exact test, using GraphPad statistical software (http://www.graphpad.com/quickcalcs/contingency2).

## Results and Discussion

Antibodies to *Leishmania* spp. were detected in 34 of 146 brown rats (23.3%) (CI 95% from 16.70 to 30.99). The serological results according to parish, sex and age are presented in Table-1. No significant differences were found between sexes and young or adult rats. The prevalence between parishes (St. George and St. David) was also not significant.

**Table-1:** Prevalence of visceral leishmaniasis in brown rats from Grenada according to parish, gender, and age.

Parish	Tested	Positive (%)	Male		Female		Young		Adult	
			
			Tested	Positive (%)	Tested	Positive (%)	Tested	Positive (%)	Tested	Positive (%)
St. Georges	71	14 (19.7)	35	6 (17.1)	36	8 (22.2)	12	2 (16.6)	58	12 (20.6)
St. David	75	20 (26.6)	45	14 (31.1)	30	6 (20)	8	2 (25.0)	68	18 (26.4)
Total	146	34 (23.3)	80	20 (25.0)	66	14 (21.2)	20	4 (20.0)	126	30 (23.8)

There are limited studies on leishmaniasis in *R. norvegicus*. Di Bella *et al*. [16] reported a 33.3% seropositivity in *R. norvegicus* in Italy using indirect immunofluorescent techniques. In the same study, they found 57.55% seropositivity by IFA and 45% by molecular technique in *Rattus rattus*. A much lower prevalence of *Leishmania* spp. in 1 of 16 (6.25%) in *R. norvegicus* in Greece was found by Papdogiannakis *et al*. [17]. They used molecular techniques for the diagnosis. A similar prevalence (23.3%) was reported by Caldert *et al*. [18] in *R. rattus* in Brazil using ELISA test. However, they found a lower prevalence with indirect fluorescent antibody test (IFAT) (3.3%) and polymerase chain reaction (PCR) (7.1%). The results of previous researchers show that variation in the prevalence of *Leishmania* spp. in rats (*R. rattus and R. norvegicus*) differs with different diagnostic techniques and in different geographic regions.

VL can be diagnosed by cytologic or histopathologic identification of organisms, isolation of amastigotes, detection of serum antibodies, or PCR [19]. Weese *et al*. [20] recommended serological testing as the main component for the diagnosis of leishmaniasis. Previous serological studies of leishmaniasis in wildlife successfully used the same ICT utilized in the present study [14,15]. The ICT used in this study is based on recombinant antigens, and it is considered an ideal diagnostic tool for leishmaniasis. Several studies [21,22] using the recombinant antigen-based ICT demonstrated high specificity compared to traditional serological tests.

We did not observe any clinical signs of leishmaniasis in brown rats (*R. norvegicus*) under study. Milena *et al*. [23] reported no apparent clinical signs of the disease in *R. rattus* in Turkey, although rats harbored live parasites capable of infecting sand flies. Similarly, Ashford [24] reported no clinical signs in *R. norvegicus* but maintained longer infectivity for sand fly vectors. In other studies [14,25], clinical signs were also not reported in rats positive for parasites.

Caldert *et al*. [18] reported the incidence of leishmaniasis more frequently in adults compared to young rats, using ELISA. Their observations were in *R. rattus*. In the same study, authors reported that the positive proportion of female rats was higher than that of male rats taking into account all three diagnostic methods (ELISA, IFAT, and PCR). In our study, we did not find statistically significant differences in the age and sex of rats. Further, research involving a higher number of rat samples might answer this difference in sex and age.

## Conclusion

This is the first report of antibodies to VL in brown rats (*R. norvegicus*) from Grenada. The seroprevalence of VL found in the present study suggests that rats are frequently exposed to the parasite in Grenada. Although there is no report of human leishmaniasis in Grenada, the presence of moderate prevalence of antibodies against *Leishmania* spp. in *R. norvegicus* on the island could suggest rats may play a role in the transmission of VL to humans. Rats live in close proximity to humans, and they could play a role in the epidemiology of the parasite in Grenada and the Caribbean nations. Therefore, regular active surveillance for *Leishmania* spp. in rats and phlebotomine sand flies in Grenada is warranted. The results show that rats (*R. norvegicus*) in Grenada are exposed to *Leishmania* spp. The rats could play an important role in the transmission of leishmaniasis to humans and other animals in Grenada.

## Authors’ Contributions

RNS planning and overseeing of the research project and manuscript writing; KT helped in trapping the rats, anesthesia, and collection of blood; and ARD and NC performing ICT, interpretation of data, and review of the manuscript. All authors read and approved the final manuscript.

## References

[1] Jeronimo S.M.B, de Quiroz Sousa A, Pearson R.D, Mandell G.L, Bennett J.E, Dolin R (2005). *Leishmania* species: Visceral (Kala-azar), cutaneous and mucocutaneous leishmaniasis. Principle and Practice of Infectious Diseases.

[2] Bowman D.D (1999). Georgis'Parasitology for Veterinarians.

[3] Dantas-Torres F, Solano-Gallego L, Baneth G, Ribeiro V.M, Paiva-Cavalacanti M, Otranto D (2012). Canine leishmaniasis in the old and new worlds: Unveiled similarities and differences. Trends Parasitol.

[4] Gramiccia M (2011). Recent advances in leishmaniasis in pet animals: Epidemiology, diagnostics and anti-vectorial prophylaxis. Vet. Parasitol.

[5] Zeledon R (1992). Leishmaniasis in the Caribbean Islands: A review. Ann. N. Y. Acad. Sci.

[6] Liautaud B, Vignier N, Miossec C, Plumelle Y, Kone M, Delta D, Ravel C, Cabie A, Desbois N (2015). First case of visceral leishmaniasis caused by *Leishmania martiniquensis*. Am. J. Trop. Med. Hyg.

[7] Rosypal A, Tripp S, Kinlaw C, Sharma R.N, Stone D, Dubey J.P (2010). Seroprevalence of canine leishmaniasis and American trypanosomiasis in dogs from Grenada, West Indies. J. Parasitol.

[8] Kumthekar S, Chikweto A, Chawla P, Tiwari K, Gozlan J, Quentin L, Martha L.P, Paterson T, Sharma R (2014). Seroprevalence of canine leishmaniasis in owned and stray dogs from Grenada, West Indies. J. Anim. Res.

[9] Johnson R.N, Young D.G, Butler J.F, Bogaert-Diaz H (1992). Possible determination of the vector and reservoir of leishmaniasis in the Dominican Republic. Am. J. Trop. Med. Hyg.

[10] Desbois N, Pratlong F, Quist D, Dedet J (2014). *Leishmania* (*Leishmania*) *Martiniquensis* spp. *Kinetoplastida*: Trypanosomatidae, description of the parasite responsible for cutaneous leishmaniasis in Martinique Island (French West Indies). Parasite.

[11] Feliaciangeli M.D (2004). Natural breeding places of *Phlebotomine* sand flies. Med. Vet. Entomol.

[12] Panti-May J.A, Hermandez-Betancourt S, Ruiz-Pina H, Medina-Peralta S (2012). Abundance and population parameters of commercial rodents present in rural household in Yucatan, Mexico. Int. Biodeterior. Biodegradation.

[13] Burns J.M, Shreffler W.G, Benson D.R, Ghalib H.W, Badaro R, Seed S.G (1993). Molecular characterization of a kinesin-related antigen of *Leishmania chagasi* that detects specific antibody in African and American visceral leishmaniasis. Proc. Nat. Acad. Sci. U. S. A.

[14] Singh N, Mishra J, Singh R, Singh S (2013). Animal reservoirs of visceral leishmaniasis in India. J. Parasitol.

[15] Lima B.S, Dantas-Torres F, de Carvalho M.R, Marinho-Junior J.F, de Almeida E.L, Brito M.E.F, Gomes F, Brandao-Filho S.P (2013). Small mammals as host of *Leishmania* spp. in a highly endemic area for zoonotic leishmaniasis in North-Eastern Brazil. Trans. R. Soc. Trop. Med. Hyg.

[16] Di bella C, Vitale F, Russo G, Greco A, Milazo C, Aloise G, Cagnin M (2003). Are rodents a potential reservoir for *Leishmania infantum* in Italy. J. Mt. Ecol.

[17] Papdogiannakis E, Spanakos G, Kontoa V, Menaunos P.G, Tegos N, Vakalis N (2010). Molecular detection of *Leishmania infantum* in wild rodents (*Rattus norvegicus*) in Greece. Zoonoses Public Health.

[18] Caldert E.T, Freire R.L, Ferreira F.P, Regffolo B.B, Mareze M, Garcia J.L, Bregono R.M, Navarro I.T (2017). *Leishmania* in synanthropic rodents *(Rattus rattus*): New evidence for urbanization of *Leishmania amazonensis*. Rev. Bras. Parasitol. Vet.

[19] Edward B.B (2002). Leishmaniasis in North America. Emerg. Vector Borne Zoonotic Dis.

[20] Weese Scott J, Andrew S.P, Maureen E.C.A, Martha B.F (2011). Parasitic diseases. Companion Animal Diseases.

[21] Otranto D, Paradies P, Sasanelli M, Spinelli R, Brandonisio O (2004). Rapid immune chromatographic test for serodiagnosis of canine leishmaniasis. J. Clin. Microbiol.

[22] Rosypal A.C, Troy G.C, Duncan R.B, Zajac A.M, Lindsay D.S (2005). Utility of diagnostic tests used in diagnosis of infection in dogs experimentally inoculated with a North American isolate of *Leishmania infantum*. J. Vet. Intern. Med.

[23] Milena S, Jan V, Luc N, Peter V (2003). *Leishmania tropica* in the black rat (*Rattus rattus*): Persistence and transmission from asymptomatic host to sand fly vector *Phlebotomus sergenti*. Microbes Infect.

[24] Ashford R.W (1996). Leishmaniasis reservoir and their significance in control. Clin. Dermatol.

[25] Akhavan A.A, Mirhendi H, Khamesipour A, Alimohammadian M.H, Rassi Y, Bates P, Kamhawi S, Valenzuela J.G, Arandian M.H, Abdoli H, Jalali-zand N, Jafari R, Shareghi N, Ghanei M, Yaghoobi-Ershadi M.R (2010). *Leishmania* species detection and identification by nested PCR assay from skin samples of rodent reservoirs. Exp. Parasitol.

